# Reversibility of acrocyanosis and improvement of capillaroscopic pattern in a patient with polycythemia vera treated with ruxolitinib: a case report

**DOI:** 10.1007/s44313-024-00053-3

**Published:** 2025-01-30

**Authors:** Angelo Nigro

**Affiliations:** Department of Rheumatology of Lucania - UOSD of Rheumatology, “Madonna Delle Grazie” Hospital, Matera, Italy

**Keywords:** Polycythemia Vera, Acrocyanosis, Capillaroscopy, Ruxolitinib, Microcirculation

## Abstract

**Purpose:**

To report the clinical course and nailfold capillaroscopic changes in a patient with polycythemia vera (PV) and acrocyanosis, underscoring the potential of ruxolitinib to modulate microvascular dysfunction associated with myeloproliferative neoplasms.

**Methods:**

We present the case of a 78-year-old woman with PV who developed pronounced acrocyanosis and abnormal nailfold capillaroscopic findings during treatment with hydroxyurea. Due to suboptimal symptom control and hematological side effects, the therapy was switched to ruxolitinib. Clinical assessments and nailfold capillaroscopy were conducted before and after the therapeutic change.

**Results:**

Following the initiation of ruxolitinib, the patient experienced marked clinical improvement, including resolution of acrocyanosis and normalization of hematologic parameters. Follow-up capillaroscopy revealed a significant reduction in microvascular abnormalities, with restored architectural organization and only mild residual capillary tortuosity.

**Conclusion:**

Ruxolitinib may offer dual therapeutic advantages in PV by effectively managing the hematologic burden of disease and improving microvascular symptoms such as acrocyanosis. Nailfold capillaroscopy may serve as a valuable non-invasive tool for assessing and monitoring microcirculatory involvement in patients with myeloproliferative neoplasms.

## Introduction

Polycythemia vera (PV) is a chronic myeloproliferative neoplasm (MPN) characterized by erythrocyte overproduction, and is often accompanied by leukocytosis and thrombocytosis. The resulting elevated hematocrit levels lead to hyperviscosity, which predisposes patients to thrombotic events, microcirculatory disturbances, and capillaroscopic alterations. Acrocyanosis is a frequently observed clinical manifestation of PV and is typically attributed to impaired microvascular perfusion and increased blood viscosity [1]. Nailfold capillaroscopy is a non-invasive diagnostic technique that provides insights into the microcirculatory system and allows for longitudinal assessment of treatment response [2]. This report presents a case of PV in which there was significant clinical and capillaroscopic improvement following a therapeutic shift from hydroxyurea (oncocarbide) to ruxolitinib. The change in treatment regimen resulted in a marked reduction in acrocyanosis and normalization of capillaroscopic findings, suggesting the beneficial role of ruxolitinib in addressing PV-induced vascular pathology.

## Case report

A 78-year-old female initially presented to our clinic in 2021 with pronounced hand acrocyanosis. The patient had been diagnosed with PV in 2015, characterized by elevated red blood cell counts (RBC 8.2 × 10^12^/L), normal white blood cell counts (WBC 6.5 × 10^9^/L), normal platelet counts (PLT 350 × 10^9^/L), elevated hematocrit (Hct 50%) and hemoglobin (Hgb 17 g/dL), and the presence of a JAK-2 gene mutation, identified via molecular biology analysis (RT-PCR technique) on peripheral blood sampling [1]. After the diagnosis, she was treated with hydroxyurea. Phlebotomy was initially attempted; however, after the first few sessions, the patient refused further phlebotomy because of logistical difficulties, as she lived in a rural area.

**Fig. 1:**
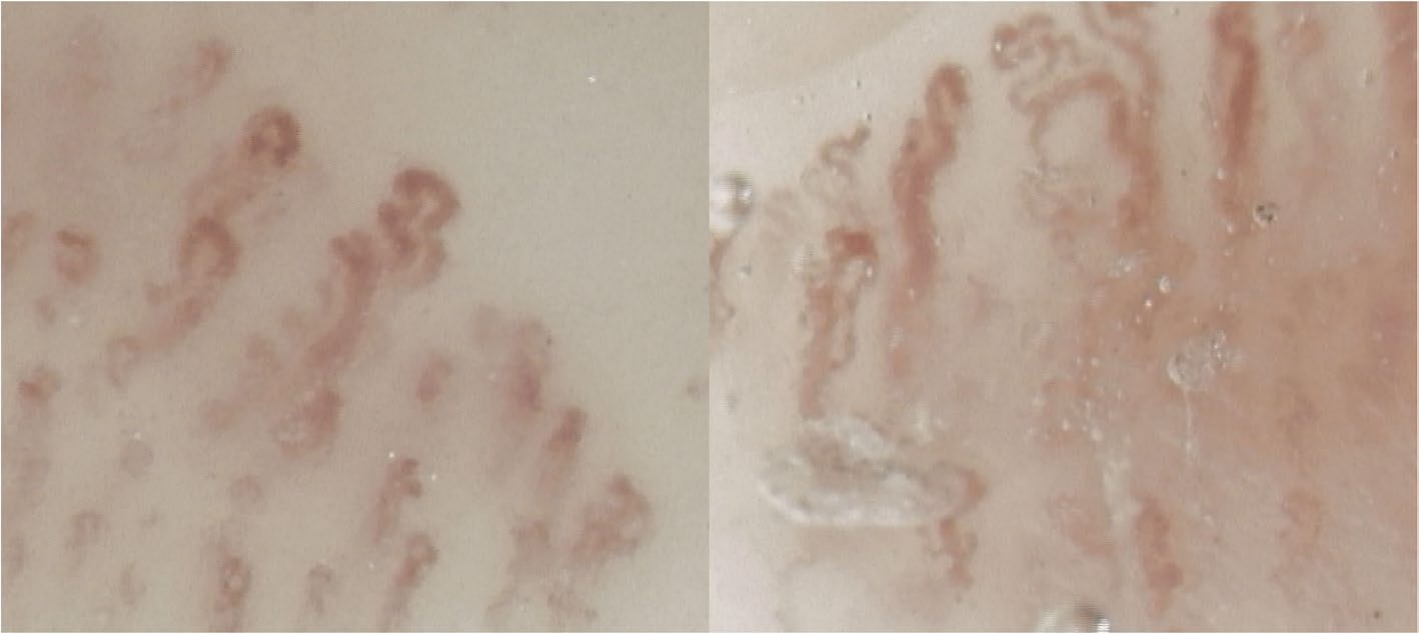
**A** and **B** Capillaroscopic images from 2021 revealing pronounced capillary tortuosity accompanied by mild architectural disorganization

**Fig. 2:**
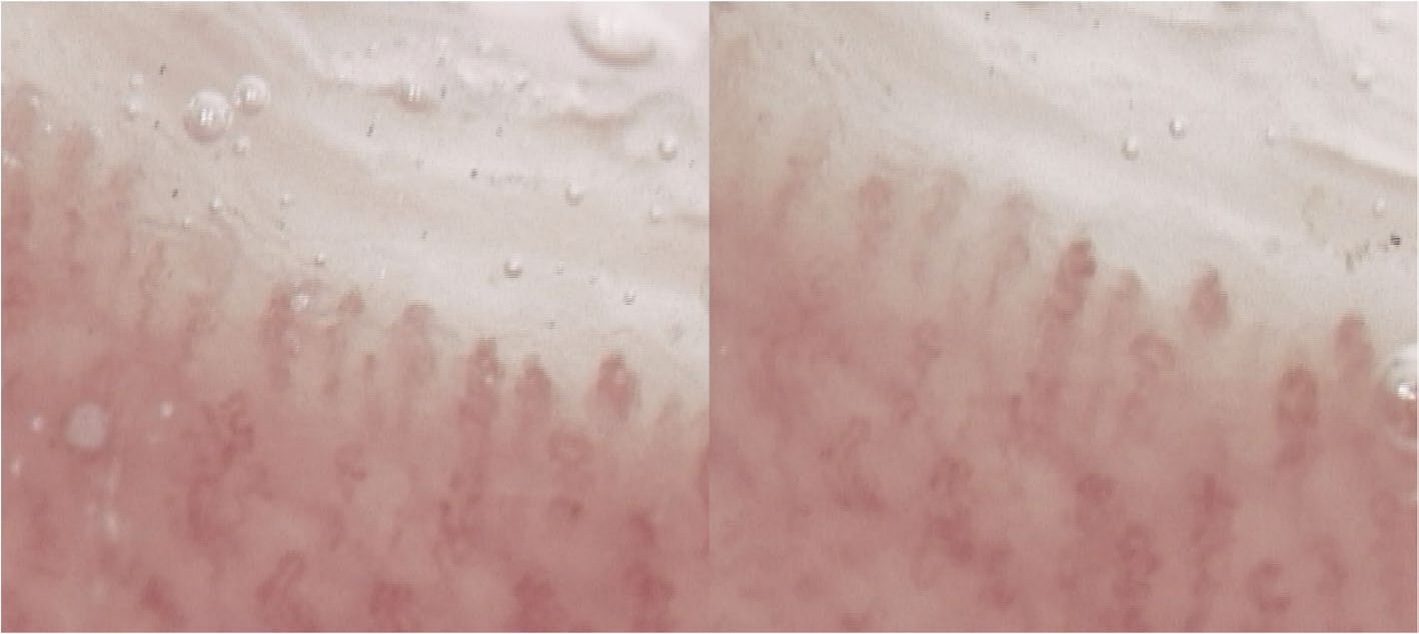
**A** and **B** Capillaroscopic images from 2024 showing a marked reduction in capillary tortuosity, with the capillary architecture nearly normalized

Nail fold capillaroscopy conducted in 2021 (Fig. 1A and B) revealed an “abnormal pattern” marked by architectural disarray and pronounced capillary tortuosity. In 2023, the therapeutic regimen was changed to include ruxolitinib, a selective JAK1/2 inhibitor, owing to the suboptimal control of symptoms and hematologic side effects, including cytopenia. Several months after initiating ruxolitinib, significant clinical improvement in acrocyanosis was observed, along with stabilization of hematologic parameters. Specifically, the blood test results showed normalization of RBC (5.0 × 10^12^/L), WBC (8.0 × 10^9^/L), PLT (350 × 10^9^/L), hematocrit (Hct 46%), and hemoglobin (Hgb 12 g/dL). A follow-up capillaroscopy conducted in October 2024 (Fig. 2A and B) demonstrated substantial normalization of the microcirculatory architecture. Residual capillary tortuosity persisted, and architectural disarray was no longer present.

## Discussion

Ruxolitinib, a potent JAK1/2 inhibitor, is approved for patients with PV who are resistant, intolerant, or experience significant adverse effects from conventional cytoreductive therapies such as hydroxyurea [3]. Its mechanism of action involves inhibition of the JAK-STAT pathway, which plays a critical role in the pathophysiology of PV by modulating cytokine-mediated processes that drive hematopoiesis. In addition to its hematological effects, ruxolitinib has demonstrated anti-inflammatory properties, likely contributing to improved microvascular function, reduced thrombotic risk, and enhanced hematologic stability [4].

This case highlights the dual therapeutic efficacy of ruxolitinib in mitigating both erythrocytosis and microcirculatory dysfunction in PV. The observed capillaroscopic improvements post-transition to ruxolitinib indicated a vasoprotective effect, likely mediated by the suppression of pro-inflammatory cytokines and the alleviation of endothelial dysfunction. Nail fold capillaroscopy, a critical diagnostic tool in rheumatology for conditions such as systemic sclerosis in which microangiopathy is a defining feature, could serve as an insightful method for assessing microvascular alterations in diseases such as PV. In this context, capillaroscopy was instrumental in the longitudinal monitoring of the patient's microvascular response to therapy, thereby providing a clear visualization of the clinical improvements achieved [5].

The findings of this case underscore the need for a holistic treatment approach for PV, in which both hematological parameters and microvascular function are continuously assessed and managed. Further studies are required to elucidate the specific mechanisms by which ruxolitinib modulates microcirculatory disturbances and evaluate its potential role in managing other MPNs with microvascular complications.

## Conclusion

This case demonstrates a significant improvement in acrocyanosis and capillaroscopic abnormalities in a patient with PV treated with ruxolitinib. These findings suggest that ruxolitinib may play a dual role in controlling erythrocytosis and managing microvascular complications associated with PV, highlighting its potential as a comprehensive therapeutic agent for MPNs, particularly in patients with microvascular involvement. Capillaroscopy may be used as a diagnostic tool for assessing microcirculatory alterations in hematological disorders such as PV, and for evaluating the effects of therapeutic interventions on microvascular integrity and function under these conditions.

## Data Availability

No datasets were generated or analysed during the current study.
